# Preferences of young physicians at community hospitals regarding academic research training through graduate school: a cross-sectional research

**DOI:** 10.1186/s13104-016-2036-0

**Published:** 2016-04-21

**Authors:** Noriaki Kurita, Minoru Murakami, Sayaka Shimizu, Junji Kumasawa, Teruhisa Azuma, Yuki Kataoka, Shungo Yamamoto, Shingo Fukuma, Yosuke Yamamoto, Shunichi Fukuhara

**Affiliations:** Department of Innovative Research and Education for Clinicians and Trainees (DiRECT), Fukushima Medical University Hospital, 1 Hikarigaoka, Fukushima, 960-1295 Japan; Department of Healthcare Epidemiology, Graduate School of Medicine, Kyoto University, Kyoto, Japan; Department of General Medicine, Shirakawa Satellite for Teaching And Research (STAR), Fukushima Medical University, Fukushima, Japan; Department of General Internal Medicine, Tenri Hospital, Nara, Japan; Center for Innovative Research for Communities and Clinical Excellence (CiRCLE), Fukushima Medical University, Fukushima, Japan; Institute for Advancement of Clinical and Transitional Science (IACT), Kyoto University Hospital, Kyoto, Japan

**Keywords:** Enrolling in graduate schools, Community hospitals, Young physicians, Cross-sectional study

## Abstract

**Background:**

Desire to attend graduate school for academic research training following the mandatory two-year clinical internship is unknown among young Japanese physicians who work at community hospitals after their internship. The aim of this study is to determine opinions and factors regarding pursuing higher education through graduate school among young physicians who work at community hospitals after their two-year internship.

**Methods:**

This cross-sectional survey was conducted among young physicians working at community hospitals after their two-year internship. We examined the percentage of young physicians considering higher education through graduate school, the planned timing and field of enrollment among those wanting to enroll, and reasons for not continuing their education among those with no such plans. The association between desire to enroll in graduate school and background characteristics was examined using modified least-squares regression to estimate proportion difference.

**Results:**

Among 127 (73.2 % internal medicine specialists, median age 30 years) physicians in 33 hospitals, 71 (55.9 %) stated that they wished to enroll in graduate school. The most frequently reported timing was 7–8 years after graduation from medical school. Those who stated no desire to attend graduate school cited concerns about the quality of training or not having enough knowledge to choose an appropriate laboratory or field, among other reasons. Increased number of years since graduating medical school [adjusted proportion difference (PD) −6.0 %, 95 % confidence interval (95 % CI) −9.8 to −2.3 %], being a woman with children [adjusted PD −53.4 %, 95 % CI −87.3 to −19.5 % (vs. a man not having children)], and completing their two-year internship at both university and community hospitals [adjusted PD −40.3 %, 95 % CI −72.5 to −8.0 % (vs. internship only at community hospitals)] were associated with a reduction in desire to enroll in graduate school.

**Conclusions:**

We identified a growing trend in desire among young physicians to attend graduate school. Attracting those young physicians who express no desire to attend graduate school, however, will require establishment of more flexible graduate school programs which address their concerns.

## Background

Physician scientists (physicians who perform research) are expected to contribute to innovation in the medical field by applying perspectives gained in clinical practice to their research [1]. However, Japan is one of many countries facing a decline in the number of physicians choosing to participate in research. Some specialists cite the 2004 introduction of the mandatory two-year clinical internship, which triggered a decrease in the number of community hospital-working young physicians returning to universities, with most instead opting to stay on at their hospitals, as one of several reasons for this trend. Before the implementation of this system, the typical career path for potential physician scientists involved enrolling in graduate school immediately following graduation from medical school or completion of their residency program and earning Ph.D. through basic research [2, 3]. Given that few programs provide Ph.D. curricula at the undergraduate level (i.e. M.D.-Ph.D. course) among medical schools in Japan, nationwide implementation of the mandatory two-year residency system resulted in an increase in the proportion of young physician working at community hospitals. When and what proportion of young physicians choose to enter graduate school after completing the two-year residency program is therefore gaining increasing importance in the education and generation of physician scientists.

A survey conducted by the Ministry of Health, Labor and Welfare targeting physicians in the second year of their 2-year internship [4] found that the proportion of respondents who wished to earn a doctorate was 30.9 % at community hospitals, which was lower than the proportion at university hospitals (40.7 %) but still respectable. Young physicians who spend their two-year internship working at university hospitals are likely to seek higher education through graduate school to earn a Ph.D. following their internship, as this route is typically recommended by university departments. However, desire to attend graduate school following an internship is unknown among young physicians who work at community hospitals after their internship. Clarification of this point may facilitate mapping out an ideal career trajectory for community hospital physicians working in current medical education system to become physician scientists, as community hospital physicians with lengthy experience can generate a number of clinical questions that can be tested through scientific research, if properly designed.

Here, we surveyed young physicians working at community hospitals after their two-year internship regarding desire to seek higher education through graduate school and factors influencing their decision regarding subsequent education.

## Methods

### Study design and participants

A cross-sectional online survey was performed from July to August 2013 among young physicians (3–10 years after graduation) who had completed their mandatory two-year internship after graduation and were currently working at community hospitals (regardless of internship workplace) but had not attended graduate school. The method of sampling was convenience sampling. Young physicians working at community hospitals, including major hospitals in Japan offering internship program (total 33 hospitals, 127 physicians), participated in this survey.

### Ethics, consent and permissions

The study protocol was approved by a central ethics committee at Institute for Health Outcomes and Process Evaluation Research, Kyoto, Japan (No. 201301). The study was conducted in accordance with the Declaration of Helsinki and the ethical guidelines for epidemiological research in Japan [5]. Consent to participate in this study was obtained when participating physicians answered to this survey.

### Items asked in this survey

The main outcome of this study was expressing a desire to enroll in graduate school and was defined as “present” or “absent” if physicians answered “yes” or “no” to the Japanese question, “Would you, in the future, like to enroll in graduate school?”, respectively.

Participants were asked to describe their background characteristics, and the proportion of physicians wishing to enroll in graduate school was calculated. Those who expressed a desire to enroll were then asked to describe the ideal timing for enrollment and target field of research. Those who expressed no desire to enroll were alternatively asked to explain the reasons for their decision (multiple responses accepted).

### Statistical analyses

For descriptive statistics, continuous variables were summarized using median, 10 and 90th percentiles. Categorical variables were summarized using frequency and proportions. To determine the relationship between background characteristics and wish to enroll, the adjusted proportion difference of physicians who wished to enroll was estimated using modified least-squares regression [6]. Statistical significance was defined as P < 0.05. Statistical analyses were conducted using STATA ver. 12 (StataCorp College Station, TX, USA).

## Results

Background characteristics of respondents are shown in Table 1. The median age and number of years after graduation from medical school were 30 and 5, respectively. Regarding field of specialization, 73 % specialized in internal medicine (includes those in training to become board-certified specialists). The proportion of physicians wishing to enroll in graduate school was 55.9 % (71 physicians), and the majority of these individuals (50.7 %, Table 2) wished to enroll 7–8 years after graduating medical school. In terms of research field, a majority of respondents expressing interest in enrolling (30 physicians, 42.3 %) wished to specialize in clinical research exclusively.

**Table 1:** Respondents’ basic information

	All (127 respondents)
Number of years since graduation from medical school	
3	24 (18.9)
4	26 (20.5)
5	21 (16.5)
6	18 (14.3)
7	9 (7.1)
8	6 (4.7)
9	8 (6.3)
10	15 (11.8)
Age, years^a^	
Median	30
10–90th percent point	27–35
Gender, n (%)	
Male	95 (74.8)
Female	32 (25.2)
Marital status, n (%)	
Single	74 (58.3)
Married	53 (41.7)
Have children, n (%)	
No	98 (77.2)
Yes	29 (22.8)
Field of specialization (including those in the training period), n (%)	
Internal medicine (includes organ specialties)	93 (73.2)
Pediatrics	9 (7.1)
Emergency medicine/intensive care	7 (5.5)
Surgery (includes organ specialties)	5 (3.9)
Family medicine	4 (3.1)
Gynecology	3 (2.4)
Urology	2 (1.6)
Anesthesiology	2 (1.6)
Radiology	2 (1.6)
Location of two-year internship	
Community hospital	102 (80.3)
University hospital	14 (11.0)
Both community and university hospitals (“*tasukigake*”)	11 (8.7)
Affiliated with university ikyoku	
No	107 (84.3)
Yes	20 (15.7)
Number of beds at the workplace hospital, n^b^	
Median	524
10–90th percent point	219–1026

^a^Data was missing from one respondent

^b^Data were missing from three respondents

**Table 2:** Desirable timing for enrolling in graduate school and choice of field

	All (N = 71)
Desirable timing for enrolling in graduate school, year	
5–6 years post-graduation	17 (23.9)
7–8 years post-graduation	36 (50.7)
9–10 years post-graduation	9 (12.7)
11–12 years post-graduation	6 (8.5)
13 years or more post-graduation	3 (4.2)
Choice of research field at graduate school	
Basic research	10 (14.1)
Clinical research	30 (42.3)
Both basic research and clinical research	17 (23.9)
Research that combines basic research and clinical research	9 (12.7)
Others^a^	5 (7.0)

^a^Others include medical education, organizational management, and healthcare administration

Table 3 describes background characteristics relative to desire to enroll in graduate school. Each one-year increase in number of postgraduate years was associated with a decline in proportion wishing to enroll in graduate school [adjusted proportion difference (PD): −6 %, 95 % confidence interval (CI): −9.8 % to −2.3 %]. With regard to combined influence of gender and children on decision, women with children experienced the most marked decrease in desire to enroll in graduate school versus men without children (adjusted PD −53.4 %, 95 % CI −87.3 % to −19.5 %). Compared to physicians who completed their two-year internship training at community hospitals solely, those who completed their internship at both university and community hospitals (a pattern known as “*tasukigake*”) was associated with a decrease in desire to enroll in graduate school (adjusted PD −40.3 %, 95 % CI −72.5 % to −8.0 %). Being affiliated with a university *ikyoku* (a kind of union organized by physicians in a division or department of medicine at graduate or medical school) while working at a community hospital was associated with an increase in desire to enroll in graduate school (adjusted PD 22.6 %, 95 % CI 2.6–42.5 %).

**Table 3:** Relationship between desire to attend graduate school and respondents’ background factors (n = 127)

	Number in category	Number wishing to attend graduate school (%)	Unadjusted	P value	Adjusted^a^	P value
			PD (95 % CI)		PD (95 % CI)	
Increase in postgraduate years, per year						
Every year	127		−5.0 (−8.8 to −1.2)	0.011	−6.0 (−9.8 to −2.3)	0.002
Gender and presence of children						
Men without children	71	42 (59.2)	Reference		Reference	
Men with children	24	15 (62.5)	3.3 (−20.2 to 26.9)	0.779	9.7 (−20.2 to 39.7)	0.52
Women without children	27	14 (51.9)	−7.3 (−30.3 to 15.7)	0.530	−11.6 (−32.7 to 9.6)	0.281
Women with children	5	0 (0.0)	−59.2 (−70.9 to −47.4)	<0.001	−53.4 (−87.3 to −19.5)	0.002
Marital status						
Single	74	42 (56.7)	Reference		Reference	
Married	53	29 (54.7)	−2.0 (−20.0 to 16.0)	0.823	0.5 (−22.6 to 23.5)	0.969
Location of two−year internship						
Community hospital	102	58 (56.8)	Reference		Reference	
University hospital	14	10 (71.4)	14.6 (−13.0 to 42.1)	0.297	7.7 (−20.6 to 35.9)	0.592
Both community and university hospitals (“*tasukigake*”)	11	3 (27.3)	−29.6 (−60.4 to 1.2)	0.060	−40.3 (−72.5 to −8.0)	0.015
Affiliated with university ikyoku						
No	107	57 (53.3)	Reference		Reference	
Yes	20	14 (70.0)	16.7 (−6.7 to 40.2)	0.16	22.6 (2.6 to 42.5)	0.027

*PD* proportion difference, *CI* confidence interval

Least-squares regression with robust variance was used to estimate differences in proportions of those wishing to enroll in graduate school

^a^All variables listed in Table 3 were forced into the regression model to estimate adjusted proportion differences

**Fig. 1:**
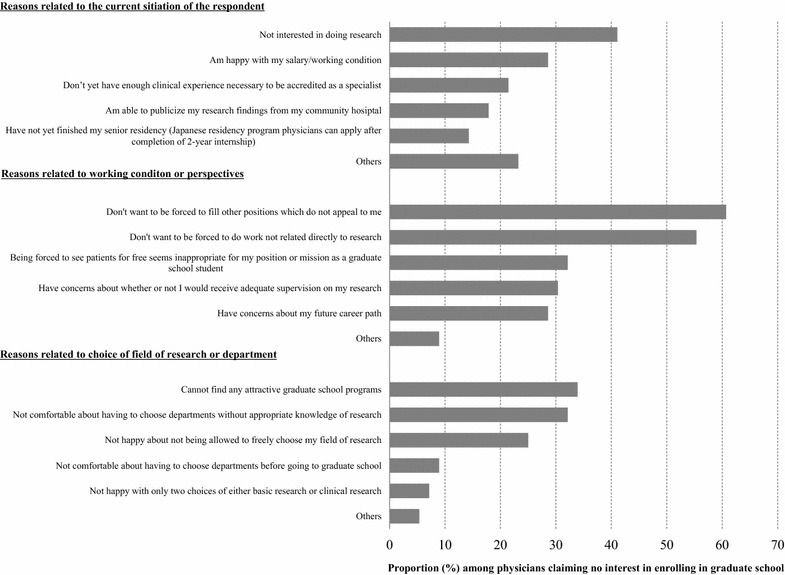
Reasons study participants did not wish to enroll in graduate school. *Gray bars* indicate the proportion of young physicians who indicated the corresponding item as a reason for not being interested in enrolling graduate school. These questions were asked to physicians who don’t wish to enroll in graduate school (N = 56). Multiple responses were accepted

Physicians who did not wish to seek higher education (56 physicians, 44.1 % of the respondents) were asked to choose why from a list of possible reasons (multiple responses accepted; Fig. 1). Thirty-four physicians (61 %) cited a fear of receiving unfavorable personnel shuffles, 31 (55 %) cited the possibility of being forced to do work irrelevant to their research and which they didn’t want to do, 23 (41 %) declared no interest in clinical research, 19 (34 %) cited a lack of any attractive graduate school programs, 18 (32 %) cited not feeling comfortable choosing a field without basic knowledge of the research involved, and 17 (30 %) worried about not having access to appropriate supervision.

## Discussion

Here, we assessed desire among young physicians working at community hospitals after their two-year internship to pursue graduate school education. We found that many young physicians working at community hospitals wish to pursue higher education and that the most frequently reported desired timing of enrollment in graduate school was 7–8 years after graduating medical school. Major reasons reported by those with no desire to pursue further education included concerns about not receiving high-quality supervision or having to choose a field without sufficient knowledge of that field. These findings may be useful in developing graduate school programs capable of attracting young physicians and stemming the decline in numbers of physician scientists.

The survey conducted by Hayashino et al. in 2008 reported that the proportion of two-year interns at community hospitals who wished to pursue graduate school education was 71.4 % [7]. The Ministry of Health, Labor and Welfare also stated in its final report, whose survey was conducted in 2005, on issues related to junior residency that the proportion of physicians in their second year of a two-year internship at community hospitals who wished to earn doctorates was 30.9 % [4], which—while lower than the proportion reported by Hayashino et al.—is still not a small number. The proportion of wishing to enroll graduate school in the present study among young physicians working at community hospitals after finishing their two-year residency was also reasonably good compared to these previous values.

Here, we found through our survey that the majority of physicians interested in seeking higher education wished to enroll in graduate school between 5 and 10 years after graduating from medical school. This length of time matches the number of years necessary to earn qualification as a board certified specialist, which agree with the findings of Hayashino et al. [7]. The previous findings of Hayashino et al. and results of our survey suggest that a number of young physicians at community hospitals may start considering higher education from their two-year internship period, stay at those hospitals for training until becoming a board certified specialist, and then choose to enroll in a graduate program as their career path. Although some specialists stress the importance of engaging in research soon after graduating medical school in order to have the best chance of success as a physician scientist, designing a graduate program which accommodates the needs of young physicians at community hospitals may also be important. To this end, the Master’s Program for Clinical Research (MCR) at Kyoto University and the Center for Innovative Research for Communities and Clinical Excellence at Fukushima Medical University already offer flexible programs catering to young physicians seeking training in clinical research [8, 9]. For example, in the MCR program, graduate students can learn theories and practical methods for conducting clinical research (i.e. formulation of answerable research questions, literature search, clinical epidemiology, biostatistics, protocol writing, and English manuscript writing) throughout the year and earn a Masters of Public Health degree. Indeed, as of fiscal year 2015, more than 400 clinical research papers have been published by physicians who completed this program, in a number of prestigious journals, including the *New England Journal of Medicine*, *Lancet*, and *JAMA* [10].

Our survey revealed several factors that might influence physicians’ decision to seek higher education. First, as more years passed after graduation from medical school, the proportion of physicians who wished to enroll in graduate school declined. One reason for this decline may be the perceived gap in knowledge necessary for research. Physicians at community hospitals tend to stay at their posts for relatively long periods of time because of strong desire to practice, thereby increasing the amount of time between graduation and any subsequent education. However, this lengthy experience in a clinical setting in and of itself positions these individuals to become talented physician scientists, as such physicians can identify research questions worth investigating by applying perspectives gained in clinical experience to their research [1, 11]. For example, during 2.5 days of clinical practice in family medicine, 1101 clinical questions were generated by 103 physicians, 20 % of which were unresolved [12]. However, these questions can be accumulated over the years by experienced physicians and transformed into valuable research questions to be tested through literature search and research protocol development. One strategy for attracting such physicians might involve a representative visiting community hospitals to inform physicians about programs such as MCR at Kyoto University which cater to physicians wishing to learn methods for clinical research in a structured environment via systematic lectures to resolve research questions encountered in daily clinical practice. Another factor influencing decision to continue one’s education is the presence of children; proportion of desire to enroll in graduate school was extremely low among female physicians with children. While our results need to be corroborated by future studies, as the present survey involved only five female physicians with children, the finding is nevertheless valuable, considering the fact that young female physicians often experience difficulty in returning to a clinical setting once they start having children. Indeed, a nationwide cohort revealed that the proportion of Japanese female physicians who take leave is higher than the proportion of those who return to work, [13] primarily due to taking leave related to childcare responsibilities [14, 15]. Proportions expressing a desire to enroll in graduate school did not markedly differ between male physicians with children and those without, suggesting that this problem is primarily restricted to female doctors. Bolstering childcare support for female doctors with children or offering distance-learning programs for home education may help ameliorate this issue of low enrollment in graduate education. Addressing such unmet needs is crucial in designing effective and inviting graduate school programs.

With regard to strengths of our study, we examined current attitudes among young physicians who had already finished their two-year internship toward enrollment in graduate school, a group that has been largely overlooked by prior surveys. The present multicenter study also covered a rather broad area of Japan, covering 33 hospitals from Kyushu to Hokkaido.

However, certain limitations to our study warrant mention. First, this study is based on convenience sampling. Physicians who cooperated in answering our questionnaire might have been interested in higher education in the first place, which could mean that the found proportion of 55.9 % wishing to enroll in graduate school is markedly higher than reality. Second, roughly 70 % of the physicians who answered the questionnaire either specialized in internal medicine or were currently being trained in internal medicine, hampering application of our findings to physicians involved in other specialties. Third, whether or not those young physicians who expressed a desire to enroll in graduate school will actually do so in the future remains to be clarified by longitudinal study. Fourth, regarding marital status, we only asked respondents whether or not they were married at the time of the survey; we were therefore unable to assess the influence of previous marriage partners in divorced individuals or that of partners in short- and long-term unmarried relationships.

## Conclusions

In summary, this study indicated that interest in pursuing higher education for academic research training is relatively common among young physicians working at community hospitals. The most frequently reported timing of enrollment for physicians wishing to attend graduate school was 7–8 years after graduating medical school. Major reasons for not wishing to pursue further education included concerns about not receiving high-quality supervision or about having to choose a field without sufficient knowledge about that field. A realistic strategy for addressing the anticipated decline in the number of physician investigators would be to design a flexible graduate school program to accommodate the growing need for young physicians who start two-year internship at community hospitals and continue to practice for clinical research, as their number is increasing. We therefore believe the intentions of young physicians regarding graduate school revealed in the present study will offer a good base for discussion.
